# Complete Genome Sequence of Bacteriophage Loca, Isolated on a Microbacterium foliorum Culture

**DOI:** 10.1128/mra.00783-22

**Published:** 2022-09-06

**Authors:** Aurod Ounsinegad, Megan Ashcraft, Emily Bliss, Dasire Brawley, Grace Clements, Austin Densmore, Alexis Gastin, Marisol Luciano, Cole Moore, Virginia Munoz, Aryana Pernarelli, Maci Pitner, Esmae Velsen, Kara Wiggam, Marlee Goppert, Dustin Edwards

**Affiliations:** a Department of Biological Sciences, Tarleton State University, Stephenville, Texas, USA; Queens College CUNY

## Abstract

Microbacteriophage Loca was extracted from a shopping cart handle swab sample in Stephenville, TX, and isolated on a Microbacterium foliorum NRRL-24224 culture. The 17,475-bp double-stranded DNA genome contains 25 predicted protein-coding genes and has >96% nucleotide identity to bacteriophages Quaker and Livingwater.

## ANNOUNCEMENT

**FIG 1:**
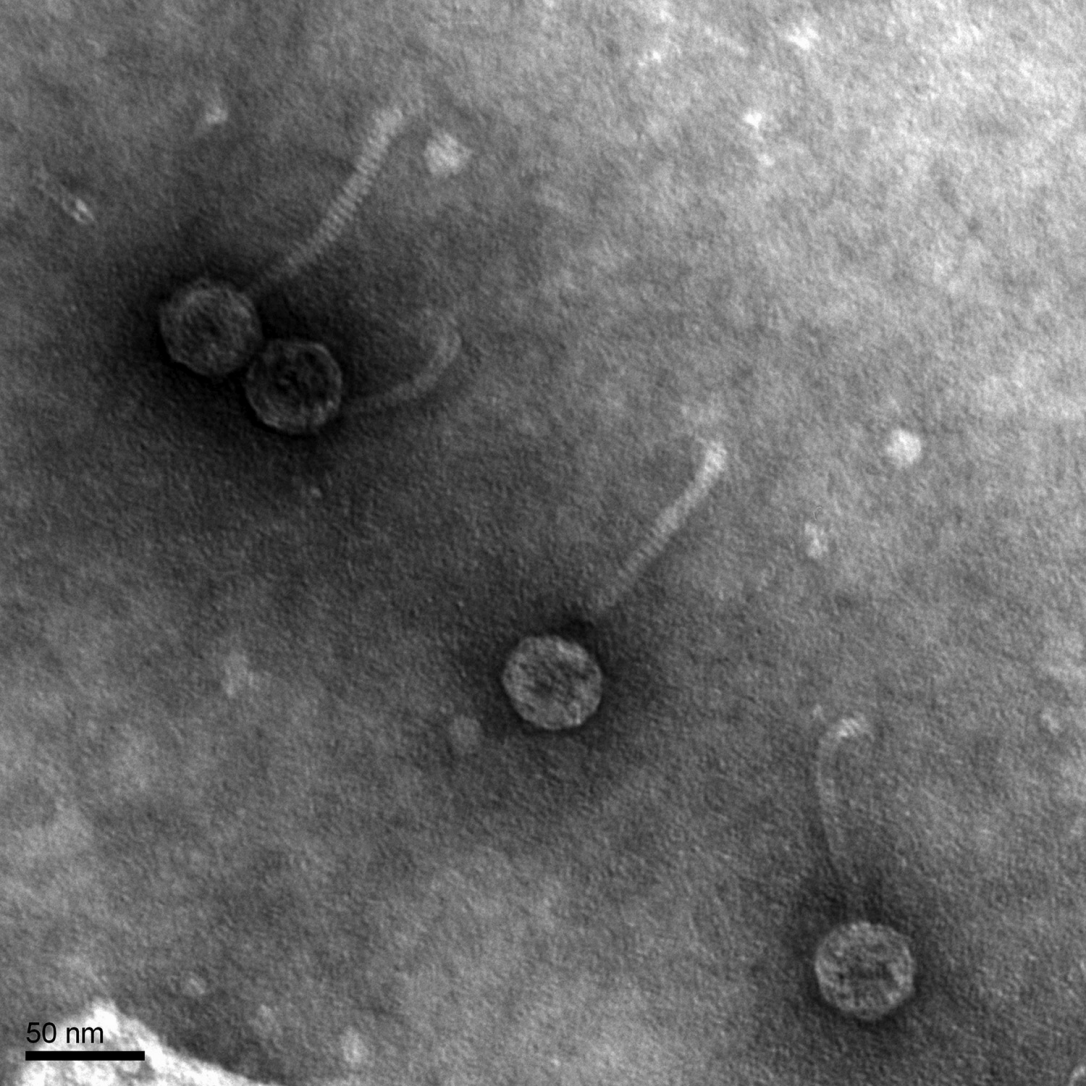
Transmission electron microscopy of microbacteriophage Loca. High-titer lysate was placed on a 300-mesh copper grid and negatively stained with uranyl acetate. Imaging with an FEI Tecnai G2 Spirit BioTWIN transmission electron microscope (NL1.160G) showed an approximate capsid diameter of 40 nm, a tail length of 105 nm (*n* = 9), and *Siphoviridae* morphology.

*Microbacterium* bacteriophages are genetically diverse and composed of several types of genomic architectures containing multiple genes of unknown function (1). To expand our knowledge of the diversity of microbacteriophages isolated from central and north Texas (2–4), we report the genome sequence of microbacteriophage Loca, collected from a swab sample of a shopping cart handle in Stephenville, TX (global positioning system [GPS] coordinates, 32.206238 N, 98.23701 W). The sample was submerged in peptone-yeast extract-calcium (PYCa) liquid medium and incubated while shaking for 2 h at 220 rpm and 29°C. The supernatant was filtered through a 0.22-μm filter and incubated with the bacterial host strain Microbacterium foliorum NRRL B-24224 in PYCa liquid medium for 6 days at 29°C (5, 6). The bacteria were pelleted and the supernatant passed through 0.22-μm filters. The filtrates were 10-fold serially diluted in phage buffer (10 mM Tris [pH 7.5], 10 mM MgSO_4_, 68 mM NaCl, 1 mM CaCl_2_, 10% glycerol) and incubated with *M. foliorum* in a soft agar overlay on PYCa agar plates for 48 h at 29°C. Bacteriophage replication formed clear, circular plaques approximately 5 mm in diameter within the soft agar overlay. Loca was isolated by two rounds of picking a single, well-separated plaque, followed by 10-fold serial dilution of the bacteriophage sample and plating it with *M. foliorum* as before. High-titer lysates were prepared by flooding “webbed” plates with phage buffer overnight at 4°C, as described in *Phage Discovery Guide* (5). Negative-staining transmission electron microscopy showed that Loca exhibited *Siphoviridae* morphology (Fig. 1), and ImageJ v1.53m (7) was used to measure an approximate tail length of 105 nm and capsid diameter of 40 nm (*n* = 9).

Genomic DNA was extracted from the high-titer lysate using a modified zinc chloride precipitation method (5, 8). The Pittsburgh Bacteriophage Institute prepared sequencing libraries using the NEBNext Ultra II DNA library prep kit (New England Biolabs, Ipswich, MA) and sequenced them using an Illumina MiSeq instrument to produce 20,276 single-end 150-bp reads. The raw reads were assembled using Newbler v2.9 to generate a single contig with 75× coverage that was checked for completeness and genomic termini using Consed v29 (9, 10). The 17,475-bp double-stranded DNA genome contains 9-nucleotide 3′ single-stranded cohesive ends (5′-CCCGCCCCA-3′) and 68.7% G+C content.

A BLASTn (11) query of the sequence against the nonredundant/nucleotide (nr/nt) database returned >96% nucleotide sequence identity to the cluster EE bacteriophages Quaker (GenBank accession number MH371111) and Livingwater (MT498040) (1). Auto-annotation with GLIMMER v3.02 (12) and GeneMark v2.5p (13) was manually refined using Phamerator (14), PECAAN, and DNA Master v5.23.3 (http://phagesdb.org/DNAMaster/). No tRNA genes were identified using Aragorn v1.1 (15) or tRNAscan-SE v2.0 (16). Putative functions for 18 of 25 predicted protein-coding genes were assigned using BLASTp (11) and HHpred (17). All tools were run with default parameters. Rightward-transcribed genes 1 to 19 encode virion structural and assembly proteins and terminase and endolysin proteins. Leftward-transcribed genes 20 to 22 encode an Lrs2-like DNA-bridging protein and two helix-turn-helix DNA binding domains. Rightward transcribed genes 23 to 25 encode a helix-turn-helix DNA binding domain and HNH endonuclease.

### Data availability.

The sequence has been deposited under GenBank accession number ON260814 and the raw reads under SRA accession number SRX14483214.

## References

[1] Jacobs-Sera D, Abad LA, Alvey RM, Anders KR, Aull HG, Bhalla SS, Blumer LS, Bollivar DW, Bonilla JA, Butela KA, Coomans RJ, Cresawn SG, D'Elia T, Diaz A, Divens AM, Edgington NP, Frederick GD, Gainey MD, Garlena RA, Grant KW, Gurney SMR, Hendrickson HL, Hughes LE, Kenna MA, Klyczek KK, Kotturi H, Mavrich TN, McKinney AL, Merkhofer EC, Moberg Parker J, Molloy SD, Monti DL, Pape-Zambito DA, Pollenz RS, Pope WH, Reyna NS, Rinehart CA, Russell DA, Shaffer CD, Sivanathan V, Stoner TH, Stukey J, Sunnen CN, Tolsma SS, Tsourkas PK, Wallen JR, Ware VC, Warner MH, Washington JM, Westover KM, et al. 2020. Genomic diversity of bacteriophages infecting Microbacterium spp. PLoS One 15:e0234636. doi:10.1371/journal.pone.0234636. 32555720 PMC7302621

[2] Lee T, Aguirre M, Andrews S, Bahr K, Ballard A, Bristerpostma M, Cox F, Dowell L, Kiker D, Lujan T, Luka S, Ramirez A, Sandoval R, Underhill K, Murphy H, Cabrera C, Edwards D. 2019. Complete genome sequence of bacteriophage Finny, isolated from a Microbacterium foliorum culture. Microbiol Resour Announc 8:e01039-19. doi:10.1128/MRA.01039-19. 31582442 PMC6776782

[3] Adams S, Spotz G, Babcock R, Butler C, Conger S, Crew M, Garcia S, Gonzalez J, Hodges J, Martinez A, Munoz S, O'Grady C, Quirl A, Sefcik K, Taylor T, Vazquez G, Cox F, Edwards D. 2022. Complete genome sequence of bacteriophage Fizzles, isolated from Microbacterium foliorum. Microbiol Resour Announc 11:e0107721. doi:10.1128/MRA.01077-21. 34989620 PMC8759393

[4] Suris A, Alvarado S, Butler T, Canales C, Castro M, Gaston J, Goppert M, Huckaby R, Laposky J, Lee J, Mullins E, Ustundag D, Zuniga J, Cox F, Edwards D. 2021. Complete genome sequence of bacteriophage IndyLu, isolated from a Microbacterium foliorum culture. Microbiol Resour Announc 10:e0107921. doi:10.1128/MRA.01079-21. 34913713 PMC8675261

[5] Poxleitner M, Pope W, Jacobs-Sera D, Sivanathan V, Hatfull G. 2018. Phage discovery guide. Howard Hughes Medical Institute, Chevy Chase, MD.

[6] Russell DA, Garlena RA, Hatfull GF. 2019. Complete genome sequence of Microbacterium foliorum NRRL B-24224, a host for bacteriophage discovery. Microbiol Resour Announc 8:e01467-18. doi:10.1128/MRA.01467-18. 30714032 PMC6357638

[7] Schneider CA, Rasband WS, Eliceiri KW. 2012. NIH Image to ImageJ: 25 years of image analysis. Nat Methods 9:671–675. doi:10.1038/nmeth.2089. 22930834 PMC5554542

[8] Santos MA. 1991. An improved method for the small scale preparation of bacteriophage DNA based on phage precipitation by zinc chloride. Nucleic Acids Res 19:5442. doi:10.1093/nar/19.19.5442. 1656393 PMC328918

[9] Russell DA. 2018. Sequencing, assembling, and finishing complete bacteriophage genomes, p 109–125. *In* Clokie MRJ, Kropinski AM, Lavigne R (ed), Bacteriophages: methods and protocols, vol 3. Springer New York, New York, NY. 10.1007/978-1-4939-7343-9_9 29134591

[10] Gordon D, Green P. 2013. Consed: a graphical editor for next-generation sequencing. Bioinformatics 29:2936–2937. doi:10.1093/bioinformatics/btt515. 23995391 PMC3810858

[11] Altschul SF, Gish W, Miller W, Myers EW, Lipman DJ. 1990. Basic local alignment search tool. J Mol Biol 215:403–410. doi:10.1016/S0022-2836(05)80360-2. 2231712

[12] Delcher AL, Harmon D, Kasif S, White O, Salzberg SL. 1999. Improved microbial gene identification with GLIMMER. Nucleic Acids Res 27:4636–4641. doi:10.1093/nar/27.23.4636. 10556321 PMC148753

[13] Besemer J, Borodovsky M. 2005. GeneMark: Web software for gene finding in prokaryotes, eukaryotes and viruses. Nucleic Acids Res 33:W451–W454. doi:10.1093/nar/gki487. 15980510 PMC1160247

[14] Cresawn SG, Bogel M, Day N, Jacobs-Sera D, Hendrix RW, Hatfull GF. 2011. Phamerator: a bioinformatic tool for comparative bacteriophage genomics. BMC Bioinformatics 12:395. doi:10.1186/1471-2105-12-395. 21991981 PMC3233612

[15] Laslett D, Canback B. 2004. ARAGORN, a program to detect tRNA genes and tmRNA genes in nucleotide sequences. Nucleic Acids Res 32:11–16. doi:10.1093/nar/gkh152. 14704338 PMC373265

[16] Lowe TM, Chan PP. 2016. tRNAscan-SE On-line: integrating search and context for analysis of transfer RNA genes. Nucleic Acids Res 44:W54–W57. doi:10.1093/nar/gkw413. 27174935 PMC4987944

[17] Zimmermann L, Stephens A, Nam S-Z, Rau D, Kübler J, Lozajic M, Gabler F, Söding J, Lupas AN, Alva V. 2018. A completely reimplemented MPI Bioinformatics Toolkit with a new HHpred server at its core. J Mol Biol 430:2237–2243. doi:10.1016/j.jmb.2017.12.007. 29258817

